# Does the presence of endometriosis cause a challenge for transvaginal oocyte retrieval? A comparison between patients with and without endometriosis

**DOI:** 10.4274/jtgga.2017.0146

**Published:** 2018-08-06

**Authors:** Işıl Kasapoğlu, Pınar Türk, Aylin Dayan, Gürkan Uncu

**Affiliations:** 1 Department of Obstetrics and Gynecology, Uludağ University School of Medicine, Bursa, Turkey

**Keywords:** Endometriosis, oocyte pickup, obesity, complication, in vitro fertilization

## Abstract

**Objective:**

The aim of the study was to compare patients with and without endometriosis regarding performance rates, difficulties, and complications associated with transvaginal oocyte retrieval (TVOR) procedures.

**Material and Methods:**

A prospective cohort study was conducted at the In Vitro Fertilization Unit of the Division of Reproductive Endocrinology and Infertility Department of a university hospital. Fifty-eight patients with endometriosis and 61 patients without endometriosis underwent TVOR procedures consecutively. Primary outcome measures were; number of needle entries per patient and performance rating defined as the total number of oocytes retrieved per vaginal needle entry. The requirement for manual compression of the abdominal wall (assistance) to reach the ovaries, procedure-related pain, and procedural complications were also evaluated.

**Results:**

The median number of needle entries through the vaginal wall per patient was comparable between the two groups (p=0.45). Performance rates were higher in the control group (p=0.001). Performance rates and total number of the needle entries through the vaginal wall were not significantly correlated with ovarian endometrioma (OMA) diameter (r=0.28; p=0.68; r=0.275, p=0.068, respectively) in the endometriosis group. Body mass index (BMI) scores were found to be correlated with the number of the needle entries and higher BMI scores were associated with higher numbers of vaginal wall punctures (p<0.001). The requirement for manual compression of the abdominal wall was significantly higher in the control group (57.4% vs 27.6%, p=0.001). A similar proportion of women needed analgesic medications after the TVOR procedure in both groups (10.3% vs 16.4%, p=0.33). Hospital readmissions for any symptoms were also comparable between the two groups (p=0.22). Three women were treated for pelvic infection, all of whom were in the endometriosis group.

**Conclusion:**

Endometriosis seems to cause a challenge for TVOR that may have reflection on individual surgeon’s performance rates for the procedure, independently from the diameter of a pre-existing OMA or ovarian adhesions. Obesity is another factor that may present a challenge for the procedure.

## Introduction

In vitro fertilization (IVF) has become the treatment of choice for many cases of infertility. Such common use of IVF has promoted ongoing development of methods to be used in each individual stage of IVF (1).

Oocyte pickup (OPU) is one of the most important stages and since the first description of follicular aspiration under transvaginal ultrasound guidance in the early 1980s, it has gained superiority because of its simplicity and because it is a successful method (2,3).

Although transvaginal oocyte retrieval (TVOR) has been accepted as a safe, straightforward, gold standard procedure, it may be associated with complications (4) and it is recommended to collect as many oocytes as possible with a minimal number of punctures (5). Procedural complications include vessel punctures, hemorrhage, injury of adjacent organs, ovarian torsion, pelvic infection, and vaginal vault bleeding (6,7). Although there are limited data on TVOR complications in the literature, serious complications are currently reported to be less than 1.5% (8). Intraabdominal hemorrhage or bleeding from vaginal puncture sites are the most common complications (9). There is limited knowledge about complications, probably as a result of underreporting.

A significant proportion of patients with endometriosis may require assisted reproductive technologies. Previous studies and meta-analyses on endometriosis have only included reproductive outcomes and have also highlighted inadequacy of data on complications that might occur in the course of IVF treatment, especially during the OPU procedure (10). It may be reasonable to suppose that TVOR can be more complex and challenging to perform in patients with endometriosis because of ovarian endometriomas or pelvic adhesions associated with endometriosis. Nevertheless, currently available data are limited to estimate complication rates and challenges associated with oocyte retrieval procedure in patients with endometriosis; further clinical studies are required in this field. Furthermore, the impact of the presence of endometriosis has not been specifically examined in the literature, especially in terms of success rates and challenges and complications associated with oocyte retrieval procedures.

In this study, we aimed to compare complications, performance rates and challenges associated with TVOR procedures between patients and without endometriosis.

## Material and Methods

This was a prospective cohort study conducted in 119 consecutive patients who underwent IVF treatment for a time period of three months (2017) at the IVF Unit of the Division of Reproductive Endocrinology and Infertility Department of a university hospital. Fifty-eight women with endometriosis and 61 without endometriosis who were body mass index (BMI)-matched, and who underwent a TVOR procedure before intracytoplasmic sperm injection (ICSI) during the same period were included in the study. Each TVOR procedure was performed by the same experienced physician. The diagnosis of endometriosis was confirmed by the demonstration of ovarian endometriomas (OMAs) either through laparoscopic surgery or using ultrasound scans.

The control group consisted of women who underwent IVF/ICSI due to male factor indications, diminished ovarian reserve, tubal factors, polycystic ovary syndrome, hypogonadotropic hypogonadism, fertility preservation, and unexplained infertility.

The study protocol was approved by the institutional Review Board and each participant provided written informed consent.

Although different ovarian stimulation protocols were used according to IVF indications, all patients received human chorionic gonadotropin (hCG) when the leading follicle(s) reached a diameter of 17 to18 mm and a TVOR procedure was performed 34-36 hours after hCG administration.

All TVOR procedures were conducted under general anesthesia with propofol and fentanyl administered to every patient. Following induction anesthesia, patients were placed in the dorsal lithotomy position; the vagina was cleaned with saline solution. Ultrasound scans were performed with a SIUI CTS-310B ultrasound machine equipped with a 3.5 to 7.5-MHz probe. A single-lumen 17-gauge aspiration needle (K-OSN-1735-A-90-US, Cook) was used in all TVOR procedures.

After the OPU procedure, patients were observed for a minimum of 2 hours. If any suspicious intraabdominal hemorrhage occurred during the TVOR procedure, patients were observed for an extra day. All patients were recommended to use 3 doses of 500 mg tablets of oral azithromycin after the TVOR procedure.

Immediately after each TVOR procedure, the surgeon filed a report consisting of objective and quantitative parameters about the details of the procedure. Ovarian adhesions were assessed using transvaginal ultrasound and adherent ovaries were described as remained unchanged after simultaneous pressure of the transvaginal ultrasound and abdominal palpation, ovaries which become directly adherent to the peritoneum of the pouch of Douglas. Procedure reports were prepared and replicated as hardcopies before starting the study. The report included a number of questions such as total number of punctures, whether abdominal manual fixation of the ovaries was necessary (abdominal compression to push the ovaries towards the pelvis by an assistant), number of mature follicles (>14 mm) counted with transvaginal ultrasound before the retrieval procedure, number of oocytes collected during the procedure, number of mature oocytes (MII) collected, whether the ovaries were adherent (ovaries at unexpected location on ultrasound, especially behind the uterus), necessity of needle passage through the uterus, necessity of increased aspiration pressure, ovarian or vaginal bleeding, method used to control bleeding (suturing/local pressure) and severe early postoperative pain (need for additional analgesia). Presence of OMA, their location (central/peripheral), and diameters of OMAs, appearance of endometrioma content inside the pipe, and necessity of needle passage through an OMA were all recorded. For all patients, postoperative clinical or laboratory evidence of an early pelvic infection were noted.

The primary outcome measures that were compared between the two groups included the number of needle entries per patient, performance rating for OPU procedures defined as the total number of oocytes retrieved per needle entry, the requirement for assistance by manual compression of the abdomen to help reach the ovaries, OPU-related pain, and procedural complications (bleeding, infection, injuries of adjacent organs).

### Statistical analysis

The Shapiro-Wilk test was used for assessing whether the variables followed normal distribution. The variables are reported as mean (±standard deviation) or median (minimum-maximum) values. According to the normality test result, intergroup comparisons were performed using the independent samples t-test or the Mann-Whitney U test. The Kruskal-Wallis test was used for comparisons involving more than two groups. Pearson’s chi-square and Fisher’s exact tests were used to compare categorical variables. Relationships between continuous variables were examined using correlation analysis. Pearson’s and Spearman’s correlation coefficients were computed to interpret normality test results. The SPSS (IBM Corp. Released 2012. IBM SPSS Statistics for Windows, Version 21.0. Armonk, NY: IBM Corp.) software was used to perform statistical analyses and a p value of <0.05 was set as the level of statistical significance.

## Results

After excluding duplicated cycles of a patient, a total of 119 OPU cycles were included in the final analysis. The study group included 58 patients with endometriosis and the control group included 61 patients without endometriosis.

The analysis of patient characteristics reveal no statistically significant differences between two groups in BMI, age, and nulliparity (p=0.17, p=0.36, and p=0.25, respectively). Significant differences were found between the two groups in serum anti-Müllerian hormone (AMH) levels, previous ovarian surgery, total gonadotropin dose administered, and ovarian adhesions; these variables are shown in Table 1. The mean serum AMH were significantly lower in the endometriosis group (p<0.01). Previous ovarian surgery and ovarian adhesion rates and total gonadotropin dose administered were also higher in the endometriosis group. All procedures were performed under general anesthesia.

Forty-five out of 58 (77.6%) patients with endometriosis had OMAs. Thirty-one (68.9%) patients had unilateral OMAs and 14 patients had bilateral OMAs (31.1%). The median diameter of the OMAs was 4 cm (2,3,4,5,6,7,8,9,10,11). In two patients, endometriomas were inadvertently punctured to gain access to the follicles lying behind.

In the control group, male factor infertility was the leading indication (n=33, 54%) for the procedure, the other factors were diminished ovarian reserve, tubal factor, polycystic ovarian syndrome, hypogonadotropic hypogonadism, fertility preservation, and unexplained infertility.

The median total number of punctures per patient was comparable between the two groups (p=0.45). Procedure performance rates, defined as the number of oocytes retrieved per needle entry, were higher in the control group (p=0.001). The need for manual assistance was significantly higher in the control group (57.4% vs 27.6%, p=0.001). A similar proportion of women needed analgesia after TVOR in the endometriosis and control groups (10.3% vs 16.4%, p=0.33). No significant intergroup difference was found in all-cause hospital readmission rates; two patients from the control group and 5 patients from the endometriosis group presented with pelvic irritation symptoms (p=0.22) (Table 2).

Conditions requiring intervention or treatment such as suturing of the vaginal wall to control bleeding, intravenous antibiotics to treat pelvic infections, and injuries to adjacent organs were accepted as complications. Complications associated with the procedure are presented in Table 3.

Vaginal wall suturing had to be performed due vaginal bleeding that was unresponsive to local pressure in a total of seven patients (5.8%). Five of these patients were in the control group (p=0.28) (Table 3). Two women in the control group had intraabdominal bleeding and were hospitalized for observation, none of whom required any additional interventions. All three patients who were readmitted due to pelvic peritoneal irritation and leukocytosis after OPU and treated for pelvic infections were in the endometriosis group. Two of these patients were those who had their OMA punctured during the TVOR procedure. One patient who presented with macroscopic hematuria was diagnosed as having a bladder injury after a cystoscopy exam. She was in the endometriosis group without OMA. The TVOR procedure of this patient was ordinary.

The number of oocytes retrieved per needle entry did not significantly correlate with OMA diameters (r=0.28; p=0.68) in the endometriosis group.

The rate of ovarian adhesions detected with ultrasound was found to be higher in the endometriosis group (p<0.01). However, no significant associations were found between ovarian adhesions and the total number of oocytes retrieved per needle entry (p=0.99).

When the results were analyzed according to BMI scores, a positive correlation was found between BMI scores and total number of needle entries and higher BMI scores (>30 kg/m^2^) were associated with higher total number of needle entries (p<0.001).

## Discussion

In this prospective study, we evaluated whether the presence of endometriosis had an unfavorable effect on TVOR procedures and we concluded that endometriosis could have an unfavorable effect on the performance (total number of oocytes retrieved per needle entry) of TVOR procedures during IVF/ICSI treatment. Furthermore, obesity was defined as another factor that may present a challenge for the procedure.

Oocyte retrieval procedures should be performed with the highest possible safety and attention because these patients are actually healthy individuals. Effectiveness and success of an oocyte retrieval procedure depends on the least amount of interference and complication rates with an adequate number of oocytes retrieved during the procedure. TVOR procedure is simple and efficient in terms of a better visualization of smaller follicles and therefore, more oocytes could be harvested (4).

Although TVOR is accepted as a safe and straightforward variations and absence of clear, unique definitions for the surgical approach makes it complicated to define certain difficulty parameters for evaluation. As a parameter that has not been evaluated previously, we evaluated the need for assistance as a difficulty parameter in our study. In endometriosis, assistance may be considered because access to the ovaries can be challenging owing to higher prevalences of adnexal adhesions. In our study, when considering the need for manual abdominal compression to fix the ovaries during OPU, the need for assistance was found to be at significantly higher rates in the control group (p=0.001), and it was correlated with BMI scores. In the assessment of the need for assistance by the prevalence of ovarian adhesions, no significant associations were found between ovarian adhesions and total number of oocytes retrieved per needle entry (p=0.99). In another study, it was suggested that, TVOR procedure might be easier to perform due to limited ovarian mobility, in the presence of adherent ovaries (9). In line with this study, higher ovarian adhesion rates in the endometriosis group (p<0.001) could explain the restricted ovarian mobility and easier oocyte collection in our study. Restricted ovarian mobility might also have reduced the need for manual fixation of the ovaries in the patients with endometriosis.

The number of oocytes retrieved, which is sign of success, was evaluated in a meta-analysis of five studies that compared the mean number of oocytes collected in patients with OMA to those in control subjects. In this meta-analysis, it was concluded that a lower number of oocytes could be collected in patients with OMA than controls (10). However, at this point, the debate about OMA and ovarian reserve continued.

In endometriomas, an important issue that has not been evaluated until now is the visible oocytes that cannot be reached and cannot be collected because of OMA. In this case, retrieved oocyte percentage (visualized follicles /retrieved oocyte number) can be evaluated. As a novel approach, in our study we evaluated the number of oocytes retrieved per needle entry, which is defined as TVOR performance. Performance rates were higher in the control group than those in the endometriosis group. However, no correlation was found between OMA diameters and number of oocytes collected (p=0.68).

Furthermore, obesity was found to be another factor that might present a challenge in reaching the ovaries in our study population. This finding was also supported by the fact that the number of needle penetrations was particularly high in patients with higher BMI (>30 kg/m^2^). Moreover, an increased number of needle entries alone may suggest that the procedure is challenging. A variety of factors were found to be responsible for the increased number of needle entries, especially in obese patients and patients with endometriosis patients in our study’s population. In patients with endometriosis, the number of needle penetrations could be increased particularly in order to avoid passing through an endometrioma while in the control group, especially in the obese patients, the prominent reason was the difficulty accessing the ovaries.

Complication rates did not increase in parallel with the increased number of needle penetrations in our study’s population. However, this is a potential issue of debate. There are no direct rational studies showing that complication rates increase as the number of needle penetrations increases, and further studies with larger sample sizes are required to provide statistical evidence to make a definitive conclusion. It is generally recommended to collect as many oocytes as possible with a minimal number of punctures (5).

The sole available meta-analysis also highlighted the lack of data about potential complications that might occur during IVF treatment and especially during the OPU procedure (10). Although there are limited data on OPU complications, notably, of those that may occur during the TVOR procedure, hemorrhages and infections have been known to be the most prominent complications with incidences ranging from 0.2% to 9% and from 0.2% to 0.6%, respectively (9,11). The most common complication was bleeding at the puncture site as a result of direct trauma (7). Simple compression is usually adequate to control local bleeding and it should be done without using a speculum. Rarely, sutures are needed to stop bleeding (8,12). In a study, it was suggested that an average blood loss of 230 mL might be considered as normal for a 24-hour period after an uncomplicated OPU. Moreover, no associations were found between blood loss and the number of follicles collected during the procedure or duration of the procedure (13). It is difficult to know and exactly foresee which patients are at risk. In our study, vaginal bleeding occurred in patients who underwent vaginal punctures at lower numbers.

Intraabdominal bleeding can be detected by a pulsatile flow and pelvic accumulation of blood. Large vessels are unlikely to be injured during ultrasound-guided procedures. However, it may not always be possible to pass through a safe area in order to avoid endometriomas and sometimes this necessitates passing through the uterus, even through the endometrium. Two patients who were followed up for intraabdominal hemorrhage were in the control group. They did not need any additional interventions. Three control patients who required uterine passage did not develop any complications.

Infections associated with TVOR were another complication reported with an incidence of 0.02% (14). Inoculation of vaginal bacteria was the most apparent elucidation. The number of vaginal punctures has been implicated as a risk factor (15). Although there are no specific trials, endometriosis and especially puncture of endometriomas has been suggested to be associated with higher infection rates (16). Our results provided further support to this suggestion because pelvic infections developed in only three patients with endometriosis in our study; one whom had bilateral endometriomas 7-8 cm in diameter and the surgeon had to puncture the endometrioma to gain access and collect follicles. Another patient who had her endometrioma punctured also developed a pelvic infection. Based on this information, dimensions and location of endometriomas may be crucial for gaining access to follicles. The third patient, who had early-stage endometriosis with an ovary adherent to the posterior wall of the uterus, underwent multiple vaginal punctures and presented with peritoneal irritation findings and leukocytosis two days after the TVOR procedure. However, the small numbers of events prevent reliable statistical comparison between the patients. Although there are some case reports supporting associations between endometriomas and infection (17), many of them failed to prove such associations (6). In a retrospective analysis of 19 patients in whom it was required to pass through an endometrioma during the procedure, no patients developed infections (18). Nevertheless, endometrioma punctures should not be encouraged and we also paid attention to avoid passing through any endometriomas. There are reports indicating that perioperative prophylactic antibiotic use may also help reduce the risk for infections (19). Without endometrioma puncture, follicular contamination with endometrioma content is unusual but possible. We observed only one case of follicular contamination with endometrioma content without cyst puncture. There are limited data on follicular contamination and its frequency has been reported as 2.8% (18).

The exact number of patients with pelvic pain who needed administration of additional analgesics therapy after OPU was comparable between the groups (p=0.33). A number of potential mechanisms could be suggested. Enlarged ovaries with multiple follicles can cause irritation.

As a much rarer complication, any accidental injury to adjacent organs is also possible and should be kept in mind (20). In a study in 2670 patients who underwent TVOR procedures, no injuries to adjacent organs were reported (9). However, there is no consensus at to whether these injuries are really so rare or simply under-recognized. We had only one patient with endometriosis who presented with gross hematuria four days after TVOR. Her TVOR procedure was performed as usual without any suspicious or unexpected finding. Bladder injury was diagnosed through a cystoscopy examination, which revealed a needle entry hole in the bladder. Although adhesions found in patients with endometriosis may raise concern, it is also suggested that TVOR may be easier due to limited ovarian mobility in case of adherent ovaries (9). Limiting the number of needle entries and careful visualization under ultrasound guidance should be kept in mind because injuries are the result of punctures. Pelvic adhesions due to endometriosis or infections are suggested to increase the risk of such injuries secondary to distorted anatomy. Although unproven, practically, it can be recommended to maintain the needle guidance in a lateral position during punctures, away from the anterior structures (21,22).

In conclusion, endometriosis could have unfavorable effects on the performance of TVOR procedure during IVF/ICSI. Also, obesity could present a further challenge for TVOR procedures with an increased number of interventions, which should be evaluated in future studies. The low prevalence of major complications may prevent reliable statistical intergroup comparisons, especially in the case of infections; the probability of false- negative results should be taken in consideration. It is worth considering a trend toward higher infectious complication rates observed in patients with endometriosis after TVOR procedures, in comparison with controls. Taking care not to puncture OMAs actually awakens the conscience, so it makes the procedure special. However, proper preoperative evaluation before contemplating TVOR is a must for all patients for the safest attention possible.

## Figures and Tables

**Table 1:**
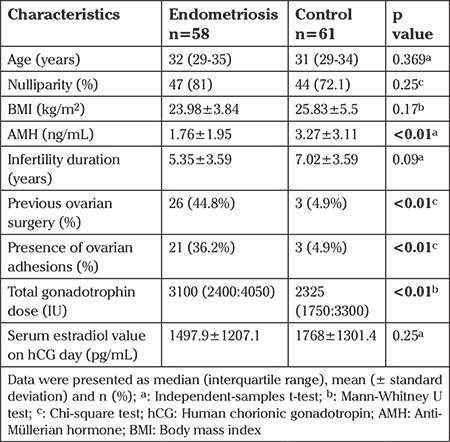
Baseline characteristics

**Table 2:**
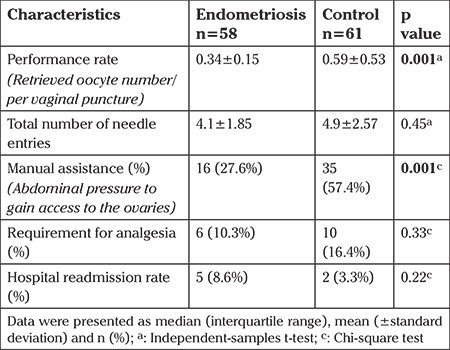
Properties of TVOR

**Table 3:**
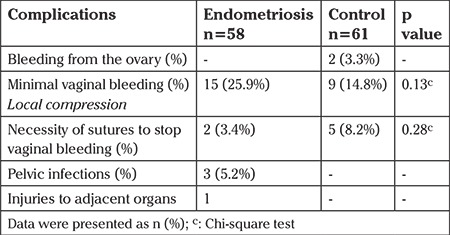
Complications

## References

[1] Centers for Disease Control and Prevention, American Society for Reproductive Medicine, Society for Assisted Reproductive Technology (2015). Assisted Reproductive Technology Fertility Clinic Success Rates Report. Atlanta (GA): US Dept of Health and Human Services.

[2] Gleicher N, Friberg J, Fullan N, Giglia RV, Mayden K, Kesky T, et al (1983). Egg retrieval for in vitro fertilization by sonographically controlled vaginal culdo-centesis. Lancet.

[3] Wikland M, Enk L, Hamberger L (1985). Transvesical and transvaginal approaches for the aspiration of follicles by use of ultrasound. Ann N Y Acad Sci.

[4] Sarhan A, Muasher SJ (2007). Surgical complications of in vitro fertilization. Middle East Fertility Society Journal.

[5] El Hussein E, Balen AH, Tan SL (1992). A prospective study comparing the outcome of oocytes retrieved in the aspirate with those retrieved in the flush during transvaginal ultrasound directed oocyte recovery for in-vitro fertilization. Br J Obstet Gynaecol.

[6] Ashkenazi J, Farhi J, Dicker D, Feldberg D, Shalev J, Ben- Rafael Z (1994). Acute pelvic inflammatory disease after oocyte retrieval: Adverse effects on the results of implantation. Fertil Steril.

[7] Evers JL, Larsen JF, Gnany GG, Seick UV (1998). Complications and problems in transvaginal sector scan-guided follicle aspiration. Fertil Steril.

[8] Ludwig AK, Glawatz M, Griesinger G, Diedrich K, Ludwig M (2006). Perioperative and post-operative complications of transvaginal ultrasound-guided oocyte retrieval: prospective study of >1000 oocyte retrievals. Hum Reprod.

[9] Bennett SJ, Waterstone JJ, Cheng WC, Parsons J (1993). Complications of transvaginal ultrasound-directed follicle aspiration: a review of 2670 consecutive procedures. J Assist Reprod Genet.

[10] Hamdan M, Dunselman G, Li TC, Cheong Y (2015). The impact of endometrioma on IVF/ICSI outcomes: a systematic review and meta-analysis. Hum Reprod Update.

[11] Maxwell KN, Cholst IN, Rosenwaks Z (2008). The incidence of both serious and minor complications in young women undergoing oocyte donation. Fertil Steril.

[12] El-Shawarby S, Margara R, Trew G, Lavery S (2004). A review of complications following transvaginal oocyte retrieval for in-vitro fertilization. Hum Fertil (Camb).

[13] Dessole S, Rubattu G, Ambrosini G, Miele M, Nardelli GB, Cherchi PL (2001). Blood loss following non- complicated transvaginal oocyte retrieval for in vitro fertilization. Fertil Steril.

[14] Andersen AN, Gianaroli L, Felberbaum R, de Mouzon J, Nygren KG;, European IVF-monitoring programme (EIM), European Society of Human Reproduction and Embryology (ESHRE) (2005). Assisted reproductive technology in Europe, 2001. Results generated from European registers by ESHRE. Hum Reprod.

[15] Serour GI, Aboulghar M, Mansour R, Sattar MA, Amin Y, Aboulghar H (1998). Complications of medically assisted conception in 3.500 cycles. Fertil Steril.

[16] Benaglia L, Somigliana E, Iemmello R, Colpi E, Nicolosi AE, Ragni G (2008). Endometrioma and oocyte retrieval-induced pelvic abscess: a clinical concern or an exceptional complication?. Fertil Steril.

[17] Younis JS, Ezra Y, Laufer N, Ohel G (1997). Late manifestation of pelvic abscess following oocyte retrieval, for in vitro fertilization, in patients with severe endometriosis and ovarian endometrioma. J Assist Reprod Genet.

[18] Benaglia L, Bermejo A, Somigliana E, Scarduelli C, Ragni G, Fedele L, et al (2012). Pregnancy outcome in women with endometriomas achieving pregnancy through IVF. Hum Reprod.

[19] Dicker D, Ashkenazi J, Feldberg D, Levy T, Dekel A, Ben-Rafael Z (1993). Severe abdominal complications after transvaginal ultrasonographically guided retrieval of oocytes for in vitro fertilization and embryo transfer. Fertil Steril.

[20] Bergh T, Lundkvist O (1992). Clinical complications during in vitro fertilization treatment. Hum Reprod.

[21] Kasapoglu I, Aslan E, Uncu G (2016). An endometriosis patient presented with gross hematuria as a complication of the oocyte pick-up procedure. J Endometr Pelvic Pain Disord.

[22] Miller PB, Price T, Nichols JE Jr, Hill L (2002). Acute ureteral obstruction following transvaginal oocyte retrieval for IVF. Hum Reprod.

